# Characteristics and care trajectories of older patients in temporary stays in Denmark

**DOI:** 10.1007/s41999-025-01209-9

**Published:** 2025-05-11

**Authors:** Hanin Harbi, Carina Lundby, Peter Bjødstrup Jensen, Søren Post Larsen, Linda Grouleff Rørbæk, Lene Vestergaard Ravn-Nielsen, Jesper Ryg, Mette Reilev, Kasper Edwards, Anton Pottegård

**Affiliations:** 1 Clinical Pharmacology, Pharmacy and Environmental Medicine, Department of Public Health, University of Southern Denmark, Campusvej 55, 5230 Odense M, Denmark; 2 Hospital Pharmacy Funen, Odense University Hospital, Odense, Denmark; 3 REAGENS, Skårup Fyn, Denmark; 4 Komponent, Copenhagen, Denmark; 5 Geriatric Research Unit, Department of Clinical Research, University of Southern Denmark, Odense, Denmark; 6 Department of Geriatric Medicine, Odense University Hospital, Odense, Denmark; 7 Centre for Suicide Research, Odense, Denmark; 8 The Research Unit in Psychiatry - Child and Adults, Psychiatry in the Region of Southern Denmark, Aabenraa, Denmark; 9 DTU Engineering Technology, Technical University of Denmark, Ballerup, Denmark

**Keywords:** Epidemiology, Morbidity, Mortality, Intermediate care facilities, Skilled nursing facilities, Aged

## Abstract

**Purpose:**

Temporary stays for patients requiring short-term care outside the home, often following hospital discharge, has gained increasing importance. This study aimed to describe the characteristics and care trajectories of older patients in Danish temporary stays to improve care delivery and patient safety.

**Methods:**

We conducted a descriptive study on a cohort of patients in temporary stays across 14 Danish municipalities from 2016 to 2023, using data from national health registries.

**Results:**

We identified 11,424 patients with a median age of 81 years (interquartile range [IQR] 73–87 years); 54% were women. Patients exhibited a high level of comorbidity, with a median Charlson Comorbidity Index of 1 (IQR 0–2), and a median of 3 hospital admissions (IQR 2–6) in the year preceding their move into temporary care. The majority (70%) transitioned to temporary stays following hospital discharge, while 30% were admitted directly from their homes. The median duration of temporary stays was 24 days (IQR 11–49 days), with 9.1% staying ≥ 90 days. Additionally, 7.0% of patients were hospitalised directly from the temporary stay facility, with a median time to hospital admission of 13 days (IQR 5–28 days). Median survival after admission to a temporary stay was 23 months (IQR 3.6–57 months). Predictors of mortality included male sex, older age, higher comorbidity burden, and increased number of hospital admissions prior to temporary stay.

**Conclusion:**

Patients in temporary stays are generally older individuals with multimorbidity and limited life expectancy. Most patients are admitted following hospital discharge, and their stays are often prolonged.

**Supplementary Information:**

The online version contains supplementary material available at 10.1007/s41999-025-01209-9.

## Key summary points

**Aim:**

To describe the characteristics and care trajectories of patients in temporary stay facilities in Denmark.

**Findings:**

Patients were generally older people with multiple chronic conditions and a median survival of about 2 years after entering the facility, most of whom entered the facility after hospital discharge. The median length of a temporary stay was 24 days, and a considerable proportion of patients was hospitalised directly or shortly after leaving the facility.

**Message:**

Patients in temporary stay facilities are typically hospital-discharged older adults with multimorbidity, limited life expectancy, prolonged stays, and high hospital admission rates.

**Supplementary Information:**

The online version contains supplementary material available at 10.1007/s41999-025-01209-9.

## Introduction

As the life expectancy increases, and population ages, more people are living with chronic diseases and disabilities. This demographic shift places significant strain on healthcare systems, which again has in most settings led to earlier hospital discharges of patients with more complex and fluctuating care needs. To manage these accelerated discharges and reduce hospital (re)admissions, many countries have reorganized their healthcare systems to strengthen patient transitions between different levels of care [1, 2]. Globally, various terms describe these transitional services, such as “intermediate care” in Europe, “subacute care”, “postacute care”, or “skilled nursing care” in the United States, “transition care” in Australia, and “transitional care” in Canada [3–5]. Intermediate care services are designed to provide short-term care to individuals who are discharged from the hospital but are not yet ready to return home, or who are at risk of hospital admission. These services can either be home-based or bed-based, with the latter involving temporary stays at designated care facilities [2, 6–8].

In Denmark, these bed-based intermediate care services are referred to as “temporary stay facilities”. Temporary stays have become increasingly important in the Danish healthcare system, especially for older patients with frailty, multimorbidity, and complex medication regimens. However, the complexity of the patients’ conditions poses significant challenges for healthcare staff, who may not always have the necessary resources or expertise to meet their specialized needs [9–14].

To improve the care and safety of patients in temporary stays, it is essential to gain deeper understanding of their characteristics and care trajectories. The aim of this study was to provide a detailed description of the characteristics, health profiles, and outcomes of patients in temporary stays in Denmark.

## Methods

We established a cohort comprising 14,978 temporary stays from 11,424 patients across 14 Danish municipalities between 2016 and 2023. The cohort was supplemented with individual-level data from Danish national administrative and health registries to describe the characteristics and trajectories of patients in temporary stays.

### Data sources

The municipalities provided data on temporary stays from January 1, 2016, to December 31, 2023, including the patient’s move-in date and move-out date, along with their Central Person Register (CPR) number, a unique personal identifier assigned by the Civil Registration System to all Danish residents since 1968 [15]. These data were linked to nationwide health registries using the CPR number. Information on hospital admissions and diagnoses was obtained from the Danish National Patient Registry, which contains data on all nonpsychiatric hospital admissions since 1977 and both psychiatric and nonpsychiatric outpatient contacts since 1995. Diagnoses have been coded according to the 10th revision of the International Classification of Diseases (ICD-10), from 1994 onwards [16]. We retrieved demographic data (age, sex, death, and migration) from the Civil Registration System [15]. For comorbidity assessment, prescription drug use was obtained from the Danish National Prescription Registry, which contains records of all prescription drugs dispensed by Danish community pharmacies since 1995 [17, 18]. We obtained information on care home admissions from a nationwide cohort of care home admissions maintained by the Danish Health Data Authority, covering admissions from 2015 onwards.

### Study cohort

Temporary stays were included if both the move-in and move-out dates occurred within the study period. We excluded temporary stays with missing or invalid CPR numbers, move-in dates, or move-out dates and those where the move-out date preceded the move-in date. Patients were required to have resided in Denmark for at least 2 years prior to their first temporary stay. For individuals with multiple temporary stays, consecutive stays with no gap between move-in and move-out dates were combined into a single continuous stay. After combining overlapping temporary stays, 21% (2,422/11,424) of the patients had more than one temporary stay during the study period. Only the first temporary stay for each patient was included in the analyses to avoid difficulties in interpretation due to non-independent observations.

### Setting

Temporary stays are provided by Danish municipalities for individuals with short-term care and support needs that cannot be met at home. These stays are available to all eligible citizens in the municipality, with no limit on their duration. Access to temporary stays is managed by the municipality. The types of stays may vary between municipalities but can include care or rehabilitation after illness or hospitalisation, as well as respite for family caregivers. The care staff may include nurses, care assistants, physiotherapists, and occupational therapists, among others. Temporary stay facilities are not required to have physicians on staff. Instead, the primary medical responsibility lies with the patient’s general practitioner or the discharging hospital. The coverage of expenses for the stay depends on its type. The so-called “acute beds” are free of charge. These beds fall under the Danish Health Act and are subject to quality standards set by the Danish Health Authority, including the requirement for round-the-clock nursing care, and accommodate the most acutely ill and unstable patients. All other types of stays fall under the Danish Social Services Act and require a small co-payment for services such as meals and laundry [11].

### Analyses

We conducted a series of analyses to describe the study cohort and patient trajectories. First, we described patient characteristics at the time of move-in overall and stratified by sex and age group (< 75, 75–84, and ≥ 85 years). Baseline characteristics included sex, age, Charlson Comorbidity Index [19], selected comorbidities (Appendix A), and number of hospital admissions in the year prior to move-in. The Charlson Comorbidity Index and comorbidities were derived from ICD-10 hospital discharge diagnoses and prescription data from the Danish National Patient Registry and Danish National Prescription Registry, using data from 10 years before baseline.

Second, we examined patient locations prior to move-in by categorizing admissions into temporary stays as following hospital discharge (defined as discharge on or the day before move-in date), from home, or from a care home, both overall and by municipality. For hospital admissions leading to temporary stays, the primary diagnosis was identified. Additionally, we calculated the median length of stay overall, by municipality, and by patient location prior to move-in. We also assessed outcomes after move-out by examining the proportion of patients who died, were hospitalised, were transferred to a care home, or were sent home directly after leaving the facility (on the move-out date or the day after). For patients with multiple outcomes, we assigned a single outcome based on the following priority: death, hospitalisation, and care home transfer. Patients who did not experience any of these outcomes were classified as having been sent home. Additionally, we examined outcomes within 30 days of move-out, considering death, hospital admission, care home transfer, or no outcome, without prioritising these events, allowing for multiple outcomes per patient. This analysis was conducted overall and separately for patients who were sent home directly after leaving the facility. In a supplementary analysis, we extended the window to 90 days. All outcome analyses were performed both overall and stratified by patient location before move-in (hospital admission or home). For patients hospitalised directly from temporary stay facilities, we assessed the median time to hospital admission, the timing of admission by hour and the day of the week, and the primary reason for admission, overall and by weekdays and weekends.

Third, to describe the mortality of patients in temporary stays, we estimated median survival after move-in and 30-day, 90-day, and 1-year survival rates, overall and by sex and age group, using the Kaplan–Meier method. We calculated odds ratios (ORs) for 30- and 90-day mortality predictors using logistic regression, with sex, age, Charlson Comorbidity Index, selected comorbidities (Appendix A), and hospital admissions in the year prior to move-in included as potential predictors.

All statistical analyses were conducted using R version 4.3.3.

## Results

We identified 14,978 temporary stays from 11,424 patients during the study period, including only the first temporary stay for each patient in our analyses (Table 1). More than half of the patients (54%) were women. The median age at move-in was 81 years (interquartile range [IQR] 73–87 years), with women being slightly older than men (median age 83 vs 79 years, *p* < 0.001). The median Charlson Comorbidity Index was 1 (IQR 0–2) and patients had a median of 3 hospital admissions (IQR 2–6) in the year prior to move-in.

**Table 1:** Baseline characteristics of patients moving into temporary stay facilities in 14 Danish municipalities from 2016 to 2023, overall and stratified by sex and age groups

	Total	Women	Men	< 75 years	75–84 years	≥ 85 years
	(n = 11,424)	(n = 6,141)	(n = 5,283)	(n = 3,394)	(n = 4,017)	(n = 4,013)
Sex						
Female	6,141 (54%)	6,141 (100%)	0 (0.00%)	1,470 (43%)	2,120 (53%)	2,551 (64%)
Male	5,283 (46%)	0 (0.00%)	5,283 (100%)	1,924 (57%)	1,897 (47%)	1,462 (36%)
Age						
Median (IQR)	81 (73–87)	83 (75–89)	79 (71–86)	68 (62–72)	80 (78–83)	90 (87–93)
< 75 years	3,394 (30%)	1,470 (24%)	1,924 (36%)	3,394 (100%)	0 (0.00%)	0 (0.00%)
75–84 years	4,017 (35%)	2,120 (35%)	1,897 (36%)	0 (0.00%)	4,017 (100%)	0 (0.00%)
≥ 85 years	4,013 (35%)	2,551 (42%)	1,462 (28%)	0 (0.00%)	0 (0.00%)	4,013 (100%)
Charlson Comorbidity Index (CCI)^a^						
Median (IQR)	1 (0–2)	1 (0–2)	2 (0–3)	1 (0–3)	2 (0–3)	1 (0–2)
0–1	5,752 (50%)	3,338 (54%)	2,414 (46%)	1,712 (50%)	1,948 (48%)	2,092 (52%)
2–3	3,921 (34%)	2,063 (34%)	1,858 (35%)	1,078 (32%)	1,441 (36%)	1,402 (35%)
≥ 4	1,751 (15%)	740 (12%)	1,011 (19%)	604 (18%)	628 (16%)	519 (13%)
Medical history of^a^						
Cancer	3,135 (27%)	1,548 (25%)	1,587 (30%)	884 (26%)	1,172 (29%)	1,079 (27%)
Chronic obstructive pulmonary disease	3,676 (32%)	2,072 (34%)	1,604 (30%)	1,171 (35%)	1,379 (34%)	1,126 (28%)
Dementia	1,428 (12%)	746 (12%)	682 (13%)	254 (7.5%)	635 (16%)	539 (13%)
Parkinson disease	477 (4.2%)	193 (3.1%)	284 (5.4%)	120 (3.5%)	248 (6.2%)	109 (2.7%)
Myocardial infarction	6,326 (55%)	3,215 (52%)	3,111 (59%)	1,515 (45%)	2,357 (59%)	2,454 (61%)
Heart failure	4,843 (42%)	2,601 (42%)	2,242 (42%)	1,209 (36%)	1,710 (43%)	1,924 (48%)
Atrial fibrillation	2,842 (25%)	1,382 (23%)	1,460 (28%)	464 (14%)	1,086 (27%)	1,292 (32%)
Stroke	3,015 (26%)	1,467 (24%)	1,548 (29%)	922 (27%)	1,169 (29%)	924 (23%)
Diabetes mellitus	2,594 (23%)	1,151 (19%)	1,443 (27%)	901 (27%)	1,010 (25%)	683 (17%)
Alcohol use disorder	735 (6.4%)	246 (4.0%)	489 (9.3%)	535 (16%)	168 (4.2%)	32 (0.80%)
Substance use disorder	558 (4.9%)	280 (4.6%)	278 (5.3%)	324 (9.5%)	172 (4.3%)	62 (1.5%)
Fall injuries	6,499 (57%)	3,902 (64%)	2,597 (49%)	1,744 (51%)	2,190 (55%)	2,565 (64%)
Hospitalizations in the year before move-in						
Median (IQR)	3 (2–6)	3 (2–5)	4 (2–7)	4 (2–7)	3 (2–6)	3 (2–5)
0–2	4,209 (37%)	2,453 (40%)	1,756 (33%)	992 (29%)	1,488 (37%)	1,729 (43%)
3–5	3,939 (34%)	2,158 (35%)	1,781 (34%)	1,152 (34%)	1,351 (34%)	1,436 (36%)
≥ 6	3,276 (29%)	1,530 (25%)	1,746 (33%)	1,250 (37%)	1,178 (29%)	848 (21%)

^a^Charlson Comorbidity Index and medical history of comorbidities were determined using the 10th revision of the International Classification of Diseases (ICD-10) hospital discharge diagnoses and prescription records from the Danish National Patient Registry and Danish National Prescription Registry, respectively, covering the 10 years prior to move-in

Most patients (70%) moved into a temporary stay facility following hospital discharge, while 30% came directly from home and 0.3% came from a care home. Patients entering from home had a higher prevalence of dementia (19% vs 9.4%, *p* < 0.001) and Parkinson disease (6.1% vs 3.4%, *p* < 0.001) but a lower median number of hospital admissions in the prior year (2 vs 4, *p* < 0.001) compared to those coming from hospital (Supplementary Table 1). The distribution of where patients came from before move-in varied across municipalities (Supplementary Table 2), with the proportion of patients coming from home ranging between 10 and 58%. Among those moving in after a hospital discharge, the three most common primary diagnoses were rehabilitation (10%), hip fracture (7.9%), and pneumonia (3.9%) (Supplementary Table 3).

**Fig. 1:**
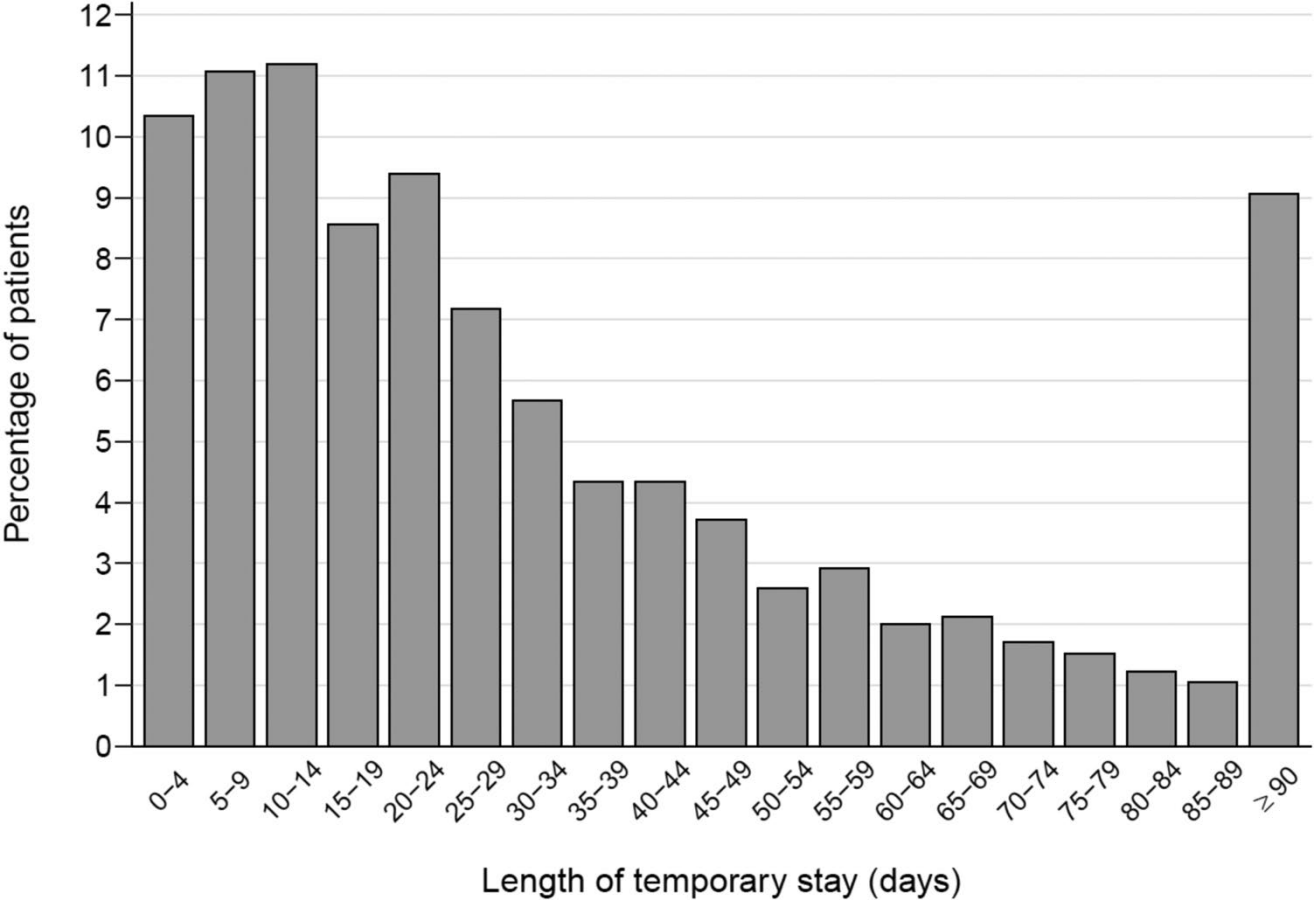
Distribution of temporary stay lengths

The median length of a temporary stay was 24 days (IQR 11–49 days), with 9.1% of patients staying for 90 days or longer (Fig. 1). Patients with stays of at least 90 days were slightly younger (median age 79 vs 81 years, *p* < 0.001) and had lower prevalences of cancer (21% vs 28%, *p* < 0.001), chronic obstructive pulmonary disease (COPD) (26% vs 33%, *p* < 0.001), and heart failure (34% vs 43%, *p* < 0.001) but higher prevalences of dementia (17% vs 12%, *p* < 0.001) and stroke (34% vs 26%, *p* < 0.001) (Supplementary Table 4). The median length of stay did not vary substantially by prior location (data not shown) or by municipality (Supplementary Fig. 1), except for one outlier municipality that showed a higher median length of stay, though with similar patient characteristics (data not shown).

Regarding patient outcomes directly after moving out of temporary stay facilities, 7.0% were hospitalised (Table 2), with a median time to hospital admission of 13 days (IQR 5–28 days). Hospitalisation rates increased gradually throughout the morning, peaking at 10 a.m., and declining later in the day, with fewer admissions during evenings and nights (Supplementary Fig. 2). Hospital admissions were most frequent on weekdays, particularly Mondays and Tuesdays, and least frequent on Sundays (Supplementary Fig. 3). The most common reasons for hospitalisation from a temporary stay facility were pneumonia (6.8%), the need for specialised palliative care (6.1%), and radiological examination (4.2%) (Supplementary Table 5, 6, 7). Additionally, 9.0% of patients died, 11% were transferred to care homes, and 73% were sent home (Table 2). Within 30 days of moving out, 16% of patients had died, 20% had been hospitalised, and 14% had been transferred to care homes (Table 2). By 90 days, these proportions had increased to 22%, 30%, and 18%, respectively. Among patients who entered temporary stay facilities after hospital discharge, a slightly higher proportion were hospitalised both directly (7.4% vs 6.4%) and within 30 days (21% vs 17%) after leaving the facility compared to those who came from their own homes (Table 2). Conversely, a lower proportion were admitted to care homes (9.8% vs 14% directly and 12% vs 17% within 30 days). Similar trends were observed with a 90-day window (data not shown).

**Table 2:** Patient outcomes directly (on the move-out date or the day after) and within 30 days after moving out of a temporary stay facility, overall and stratified by patient location before move-in (hospital admission or home). The 30-day outcome analysis was also performed separately for patients sent home directly after move-out

	Overall	Hospital admission	Home
	(*n* = 11,424)	(*n* = 7,985)	(*n* = 3,407)
Directly after temporary stay			
Death	1,032 (9.0%)	719 (9.0%)	313 (9.2%)
Hospital admission	805 (7.0%)	588 (7.4%)	217 (6.4%)
Care home	1,287 (11%)	782 (9.8%)	473 (14%)
Home	8,300 (73%)	5,896 (74%)	2,404 (71%)
Within 30 days after temporary stay			
Overall			
Death	1,881 (16%)	1,266 (16%)	613 (18%)
Hospital	2,240 (20%)	1,657 (21%)	580 (17%)
Care home	1,586 (14%)	958 (12%)	596 (17%)
None of the above	6,463 (57%)	4,603 (58%)	1,860 (55%)
Among patients sent home			
Death	535 (6.4%)	333 (5.6%)	202 (8.4%)
Hospital	1,267 (15%)	956 (16%)	311 (13%)
Care home	256 (3.1%)	148 (2.5%)	108 (4.5%)
None of the above	6,463 (78%)	4,603 (78%)	1,860 (77%)

**Fig. 2:**
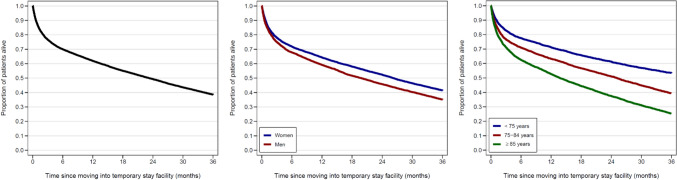
Kaplan–Meier survival curves for patients moving into temporary stay facilities in 14 Danish municipalities from 2016 to 2023, overall (left panel), stratified by sex group (middle panel), and stratified by age group (right panel)

The median overall survival after moving into a temporary stay facility was 23 months (IQR 3.6–57 months). Survival rates were 86% at 30 days, 77% at 90 days, and 62% at 1 year (Fig. 2). Men had shorter median survival than women (20 vs 26 months), as well as lower survival rates at 30 days (85% vs 87%), 90 days (75% vs 78%) and 1 year (59% vs 64%) (Fig. 2). Survival also decreased with increasing age. The median survival was 42 months for patients under 75 years, 25 months for those aged 75–84 years, and 14 months for those 85 years and older. The 30-day, 90-day, and 1-year survival rates were 91%, 83%, and 71%, respectively, for patients under 75; 86%, 77%, and 63%, respectively, for those aged 75–84 years; and 83%, 71%, and 53% for those aged 85 or older (Fig. 2).

Baseline predictors of 30-day mortality after moving into a temporary stay facility included male sex (OR 1.17, 95% confidence interval [CI] 1.05–1.32), older age (OR 1.60, 95% CI 1.37–1.87 for ages 75–84 and OR 2.29, 95% CI 1.95–2.69 for ages 85 and older compared to those under 75), higher Charlson Comorbidity Index, more hospital admissions in the year before move-in, and a history of cancer (OR 1.65, 95% CI 1.41–1.93) or heart failure (OR 1.51, 95% CI 1.34–1.71). Conversely, a history of Parkinson disease (OR 0.58, 95% CI 0.40–0.82), dementia (OR 0.72, 95% CI 0.59–0.88), and fall injuries (OR 0.77, 95% CI 0.68–0.86) was associated with decreased 30-day mortality (Table 3). A similar pattern of baseline predictors was observed for 90-day mortality (Supplementary Table 8).

**Table 3:** Baseline predictors of 30-day mortality after moving into a temporary stay facility

	OR (95% CI)
Sex	
Female	1.00 (ref.)
Male	1.17 (1.05–1.32)
Age	
< 75 years	1.00 (ref.)
75–84 years	1.60 (1.37–1.87)
≥ 85 years	2.29 (1.95–2.69)
Charlson Comorbidity Index (CCI)	
0–1	1.00 (ref.)
2–3	1.36 (1.15–1.60)
≥ 4	1.93 (1.56–2.40)
Medical history of	
Cancer	1.65 (1.41–1.93)
Chronic obstructive pulmonary disease	1.12 (1.00–1.27)
Dementia	0.72 (0.59–0.88)
Parkinson disease	0.58 (0.40–0.82)
Myocardial infarction	0.97 (0.86–1.09)
Heart failure	1.51 (1.34–1.71)
Atrial fibrillation	0.97 (0.85–1.10)
Stroke	0.82 (0.71–0.93)
Diabetes mellitus	0.91 (0.79–1.04)
Alcohol use disorder	0.83 (0.62–1.08)
Substance use disorder	0.98 (0.75–1.28)
Fall injuries	0.77 (0.68–0.86)
Hospitalizations in the year before move-in	
0–2	1.00 (ref.)
3–5	1.18 (1.02–1.35)
≥ 6	1.59 (1.38–1.83)

## Discussion

This study describes the characteristics and care trajectories of patients in temporary stays in Denmark. Our findings indicate that these patients are generally older adults with multiple chronic conditions, most of whom enter the temporary stay facility following a hospital admission. The median length of stay was 24 days, and many patients had limited life expectancy, with a substantial proportion being hospitalised directly from or shortly after leaving the facility.

The key strength of our study is the use of a large cohort of patients across multiple municipalities in Denmark, linked to highly valid nationwide health registries [15–18], which eliminates the risk of selection bias. Additionally, access to a national cohort of all care home admissions allowed us to identify the substantial number of patients who were transferred to a care home shortly after their temporary stays.

However, this study also has limitations. We lacked data on the specific types of temporary stays (e.g., rehabilitation vs respite stay), which prevented us from investigating differences in patient characteristics and outcomes across stay types, as well as the extent of reentries and transfers (from one type to another). Given that different types of stays target distinct groups (e.g., patients in rehabilitation stays typically come from hospitals, while those in respite stays often come from home) [11], this may explain the municipal differences in the distribution of patient locations prior to move-in. Additionally, our identification of reasons for hospital admission may not have been precise. We relied on primary diagnoses from the Danish National Patient Registry, which included nondiagnostic ICD-10 R and Z codes, while secondary diagnoses were not used due to their optional nature and potential for multiple entries per admission.

This is the first study to systematically describe a large cohort of patients in temporary stays in Denmark. Previous studies from a single Danish municipality, which focused on patients entering temporary stays after hospital discharge, reported similar findings in terms of sex, age, and comorbidity burden [20, 21]. However, one study observed a slightly higher 30-day mortality (17% vs 14% in our study) [21], likely because their cohort included frailer patients discharged from geriatric departments. Internationally, studies on intermediate care units in England reported a similar distribution of sex and age and a similar burden of comorbidities but noted differences in patient origins, with a lower proportion coming from hospitals (46% vs 70%) and a higher proportion from home or care homes (51% vs 30%). They also observed shorter stays (median 17 days, IQR 5–34 days vs 24 days, IQR 11–49 days in our study) and lower 1-year mortality (28% vs 38%). Similar to our findings, poorer survival was associated with increasing age, higher Charlson Comorbidity Index, and cancer [22]. In Norway, patients in municipal acute wards typically enter from home and have a median length of stay of 3 days. However, these intermediate care units are different, as they mainly target patients coming from their home to prevent hospital admissions by managing acute conditions with short-term stays in primary care, as opposed to hospital admissions [23–25].

We observed that a considerable proportion of patients were transferred to care homes directly and shortly after leaving the temporary stay facility. The higher care home admission rates observed among patients entering temporary stay facilities from home may reflect individuals awaiting long-term care placements. Generally, the morbidity and mortality profile of patients in temporary stays closely resembles that of Danish care home residents, with conditions like heart failure and cancer associated with poorer survival in care home residents [26]. Interestingly, Parkinson disease, dementia, and fall injuries were associated with lower mortality in temporary stays, possibly because these conditions prompt entry into temporary stays for less acute needs compared to conditions such as heart failure or cancer. This may also explain the differences in comorbidities by stay duration and premove-in location.

The high mortality, hospital admission, and care home admission rates observed after temporary stays underscore the vulnerability of this patient population and suggest potential challenges in the transition from hospital to community care. The fact that most patients entered temporary stay facilities after hospital discharge, and that those coming from hospital admissions had slightly higher readmission rates, may support concerns that patients are being discharged from hospitals more quickly and in less stable conditions [27–29]. These outcome rates may also reflect the performance of temporary stay facilities [27], which may lack the resources and expertise needed to address the increasingly complex and urgent care needs of these patients. This highlights the need to reevaluate current hospital discharge practices and ensure that temporary stay facilities are adequately equipped to provide the necessary care, thereby improving continuity of care and patient outcomes.

In conclusion, this study provides a comprehensive overview of the morbidity and mortality of patients in temporary stays, information essential for optimizing care transitions and ensuring better outcomes for patients. The findings underscore the complexity of caring for patients in temporary stays, a challenge that will likely increase as healthcare systems face rising demands and limited resources. It is crucial to consider patients’ health status when organizing temporary stays to ensure optimal care.

## Supplementary Information

Below is the link to the electronic supplementary material.

Supplementary file1 (DOCX 210 kb)

## Data Availability

Data on temporary stays were obtained after agreement with the individual Danish municipalities, and the national health registry data were used under license from the Danish Health Data Authority. Therefore, data cannot be made publicly available.
