# Supplementary material for: Characteristics and care trajectories of older patients in temporary stays in Denmark
Source: Eur Geriatr Med. 2025 May 11;16(4):1437–45. doi: 10.1007/s41999-025-01209-9 (PMC12378467; doi:10.1007/s41999-025-01209-9)
Supplement: Supplementary file 1 — Supplementary file1 (DOCX 210 kb) [file 41999_2025_1209_MOESM1_ESM.docx]

**Supplementary information**

**Title: Characteristics and care trajectories of older patients in temporary stays in Denmark**

**Journal:** European Geriatric Medicine

**Authors:** Hanin Harbi, Carina Lundby, Peter Bjødstrup Jensen, Søren Post Larsen, Linda Grouleff Rørbæk, Lene Vestergaard Ravn-Nielsen, Jesper Ryg, Mette Reilev, Kasper Edwards, Anton Pottegård

**Corresponding author:** Anton Pottegård, Clinical Pharmacology, Pharmacy and Environmental Medicine, Department of Public Health, University of Southern Denmark, Campusvej 55, 5230 Odense M, Denmark. Tel.: +45 28 91 33 40. Email: apottegaard@health.sdu.dk

**Supplementary Table 1**

Baseline characteristics of patients moving into temporary stay facilities in 14 Danish municipalities from 2016 to 2023, stratified by patient location prior to move-in (hospital or home)

|  | Hospital | Home |
| --- | --- | --- |
|  | (n = 7,985) | (n = 3,407) |
| Sex |  |  |
| Female | 4,369 (55%) | 1,752 (51%) |
| Male | 3,616 (45%) | 1,655 (49%) |
| Age |  |  |
| Median (IQR) | 81 (73-87) | 82 (74-88) |
| < 75 years | 2,465 (31%) | 918 (27%) |
| 75-84 years | 2,782 (35%) | 1,227 (36%) |
| ≥ 85 years | 2,738 (34%) | 1,262 (37%) |
| Charlson Comorbidity Index (CCI)^a^ |  |  |
| Median (IQR) | 1 (0-2) | 2 (0-3) |
| 0-1 | 4,109 (51%) | 1,629 (48%) |
| 2-3 | 2,675 (34%) | 1,230 (36%) |
| ≥ 4 | 1,201 (15%) | 548 (16%) |
| Medical history of^a^ |  |  |
| Cancer | 2,177 (27%) | 957 (28%) |
| Chronic obstructive pulmonary disease | 2,583 (32%) | 1,086 (32%) |
| Dementia | 752 (9.4%) | 660 (19%) |
| Parkinson disease | 269 (3.4%) | 207 (6.1%) |
| Myocardial infarction | 4,387 (55%) | 1,918 (56%) |
| Heart failure | 3,371 (42%) | 1,462 (43%) |
| Atrial fibrillation | 2,048 (26%) | 790 (23%) |
| Stroke | 2,193 (27%) | 814 (24%) |
| Diabetes mellitus | 1,814 (23%) | 773 (23%) |
| Alcohol use disorder | 542 (6.8%) | 192 (5.6%) |
| Substance use disorder | 401 (5.0%) | 156 (4.6%) |
| Fall injuries | 4,658 (58%) | 1,822 (53%) |
| Hospitalizations in the year before move-in |  |  |
| Median (IQR) | 4 (2-6) | 2 (0-5) |
| 0-2 | 2,454 (31%) | 1,730 (51%) |
| 3-5 | 3,019 (38%) | 917 (27%) |
| ≥ 6 | 2,512 (31%) | 760 (22%) |

^a^Charlson Comorbidity Index and medical history of comorbidities were determined using the 10^th^ revision of the International Classification of Diseases (ICD-10) hospital discharge diagnoses and filled prescriptions from the Danish National Patient Registry and Danish National Prescription Registry, respectively, covering the 10 years prior to move-in.

**Supplementary Table 2**

Proportion of patients moving into a temporary stay facility after hospital discharge or directly from their homes or a care home, overall and stratified by municipalities

|  | Overall | 1 | 2 | 3 | 4 | 5 | 6 | 7 | 8 | 9 | 10 | 11 | 12 | 13 | 14 |
| --- | --- | --- | --- | --- | --- | --- | --- | --- | --- | --- | --- | --- | --- | --- | --- |
|  | (n = 11,424) | (n = 975) | (n = 450) | (n = 1,060) | (n = 1,606) | (n = 632) | (n = 1,397) | (n = 349) | (n = 735) | (n = 1,001) | (n = 656) | (n = 804) | (n = 281) | (n = 1,093) | (n = 385) |
| Hospital admission | 7,985 (70%) | 800 (82%) | 287 (64%) | 507 (48%) | 1,273 (79%) | 435 (69%) | 1,175 (84%) | 250 (72%) | 464 (63%) | 532 (53%) | 467 (71%) | 489 (61%) | 253 (90%) | 896 (82%) | 157 (41%) |
| Home | 3,407 (30%) | 175 (18%) | 163 (36%) | 552 (52%) | 332 (21%) | 195 (31%) | 222 (16%) | 99 (28%) | 271 (37%) | 448 (45%) | 189 (29%) | 312 (39%) | 28 (10%) | 197 (18%) | 224 (58%) |
| Care home | 32 (0.28%) | 0 (0.00%) | 0 (0.00%) | n < 5 | n < 5 | n < 5 | 0 (0.00%) | 0 (0.00%) | 0 (0.00%) | 21 (2.1%) | 0 (0.00%) | n < 5 | 0 (0.00%) | 0 (0.00%) | n < 5 |

**Supplementary Table 3**

The 10 most frequently reported primary diagnoses (codes and names) for hospital admissions of patients discharged to temporary stay facilities

| Reason for hospital admission, diagnosis code | Reason for hospital admission, diagnosis name | Proportion of hospitalized patients, n (%) |
| --- | --- | --- |
|  |  | (n = 7,985) |
| Z508 | Rehabilitation, other | 520 (6.5) |
| S720 | Fracture of head and neck of femur | 316 (4.0) |
| J189 | Pneumonia, unspecified organism | 312 (3.9) |
| S721 | Pertrochanteric fracture | 311 (3.9) |
| Z509 | Rehabilitation, unspecified | 285 (3.6) |
| I639 | Cerebral infarction, unspecified | 223 (2.8) |
| R296 | Repeated falls | 162 (2.0) |
| E869A | Dehydration | 144 (1.8) |
| N390 | Urinary tract infection, site not specified | 113 (1.4) |
| N300 | Acute cystitis | 91 (1.1) |

**Supplementary Table 4**

Baseline characteristics of patients moving into temporary stay facilities in 14 Danish municipalities from 2016 to 2023 stratified by temporary stay length

|  | < 90 days | ≥ 90 days |
| --- | --- | --- |
|  | (n = 10,388) | (n = 1,036) |
| Sex |  |  |
| Female | 5,573 (54%) | 568 (55%) |
| Male | 4,815 (46%) | 468 (45%) |
| Age |  |  |
| Median (IQR) | 81 (74-88) | 79 (71-86) |
| < 75 years | 3,015 (29%) | 379 (37%) |
| 75-84 years | 3,666 (35%) | 351 (34%) |
| ≥ 85 years | 3,707 (36%) | 306 (30%) |
| Charlson Comorbidity Index (CCI)^a^ |  |  |
| Median (IQR) | 2 (0-3) | 1 (0-2) |
| 0-1 | 5,174 (50%) | 578 (56%) |
| 2-3 | 3,588 (35%) | 333 (32%) |
| ≥ 4 | 1,626 (16%) | 125 (12%) |
| Medical history of^a^ |  |  |
| Cancer | 2,915 (28%) | 220 (21%) |
| Chronic obstructive pulmonary disease | 3,406 (33%) | 270 (26%) |
| Dementia | 1,254 (12%) | 174 (17%) |
| Parkinson disease | 433 (4.2%) | 44 (4.2%) |
| Myocardial infarction | 5,761 (55%) | 565 (55%) |
| Heart failure | 4,492 (43%) | 351 (34%) |
| Atrial fibrillation | 2,624 (25%) | 218 (21%) |
| Stroke | 2,665 (26%) | 350 (34%) |
| Diabetes mellitus | 2,396 (23%) | 198 (19%) |
| Alcohol use disorder | 673 (6.5%) | 62 (6.0%) |
| Substance use disorder | 519 (5.0%) | 39 (3.8%) |
| Fall injuries | 5,949 (57%) | 550 (53%) |
| Hospitalizations in the year before move-in |  |  |
| Median (IQR) | 3 (2-6) | 3 (2-6) |
| 0-2 | 3,826 (37%) | 383 (37%) |
| 3-5 | 3,585 (35%) | 354 (34%) |
| ≥ 6 | 2,977 (29%) | 299 (29%) |

^a^Charlson Comorbidity Index and medical history of comorbidities were determined using the 10^th^ revision of the International Classification of Diseases (ICD-10) hospital discharge diagnoses and filled prescriptions from the Danish National Patient Registry and Danish National Prescription Registry, respectively, covering the 10 years prior to move-in.

**Supplementary Table 5**

The 10 most frequently reported primary diagnoses (codes and names) for hospital admissions of patients hospitalised directly from temporary stay facilities

| Reason for hospital admission, diagnosis code | Reason for hospital admission, diagnosis name | Proportion of hospitalized patients, n (%) |
| --- | --- | --- |
|  |  | (n = 805) |
| J189 | Pneumonia, unspecified organism | 55 (6.8) |
| Z515S | Need for specialised palliative care | 49 (6.1) |
| Z016 | Radiological examination | 34 (4.2) |
| Z039 | Observation for suspected disease or condition, unspecified | 26 (3.2) |
| R060 | Dyspnea | 23 (2.9) |
| Z508 | Rehabilitation, other | 21 (2.6) |
| A419B | Urosepsis | 14 (1.7) |
| E869A | Dehydration | 14 (1.7) |
| A499 | Bacterial infection, unspecified | 14 (1.7) |
| J159 | Bacterial pneumonia, unspecified | 11 (1.4) |

**Supplementary Table 6**

The 10 most frequently reported primary diagnoses (codes and names) for hospital admissions of patients hospitalised directly from temporary stay facilities on weekdays

| Reason for hospital admission, diagnosis code | Reason for hospital admission, diagnosis name | Proportion of hospitalized patients, n (%) |
| --- | --- | --- |
|  |  | (n = 674) |
| Z515S | Need for specialised palliative care | 49 (7.3) |
| J189 | Pneumonia, unspecified organism | 42 (6.2) |
| Z016 | Radiological examination | 34 (5.0) |
| Z039 | Observation for suspected disease or condition, unspecified | 22 (3.3) |
| Z508 | Rehabilitation, other | 21 (3.1) |
| R060 | Dyspnea | 16 (2.4) |
| A419B | Urosepsis | 12 (1.8) |
| A499 | Bacterial infection, unspecified | 12 (1.8) |
| J159 | Bacterial pneumonia, unspecified | 10 (1.5) |
| E869A | Dehydration | 9 (1.3) |

**Supplementary Table 7**

The 10 most frequently reported primary diagnoses (codes and names) for hospital admissions of patients hospitalised directly from temporary stay facilities on weekends

| Reason for hospital admission, diagnosis code | Reason for hospital admission, diagnosis name | Proportion of hospitalized patients, n (%) |
| --- | --- | --- |
|  |  | (n = 131) |
| J189 | Pneumonia, unspecified organism | 13 (9.9) |
| R060 | Dyspnea | 7 (5.3) |
| E869A | Dehydration | 5 (3.8) |
| R509 | Fever, unspecified | n < 5 |
| Z039 | Observation for suspected disease or condition, unspecified | n < 5 |
| A419 | Sepsis, unspecified organism | n < 5 |
| J449 | Chronic obstructive pulmonary disease, unspecified | n < 5 |
| N300 | Acute cystitis | n < 5 |
| Z038 | Observation for other suspected diseases and conditions | n < 5 |
| B342A | COVID-19 infection without localization | n < 5 |

**Supplementary Table 8**

Baseline predictors of 90-day mortality after moving into a temporary stay facility

|  | OR (95% CI) |
| --- | --- |
| Sex |  |
| Female | 1.00 (ref.) |
| Male | 1.15 (1.05 - 1.27) |
| Age |  |
| < 75 years | 1.00 (ref.) |
| 75-84 years | 1.58 (1.39 - 1.79) |
| ≥ 85 years | 2.34 (2.05 - 2.67) |
| Charlson Comorbidity Index (CCI) | |
| 0-1 | 1.00 (ref.) |
| 2-3 | 1.25 (1.09 - 1.43) |
| ≥ 4 | 1.93 (1.62 - 2.31) |
| Medical history of | |
| Cancer | 1.72 (1.51 - 1.97) |
| Chronic obstructive pulmonary disease | 1.16 (1.05 - 1.28) |
| Dementia | 0.71 (0.60 - 0.83) |
| Parkinson disease | 0.75 (0.57 - 0.96) |
| Myocardial infarction | 1.05 (0.95 - 1.16) |
| Heart failure | 1.39 (1.26 - 1.54) |
| Atrial fibrillation | 1.04 (0.94 - 1.16) |
| Stroke | 0.83 (0.74 - 0.92) |
| Diabetes mellitus | 1.00 (0.89 - 1.11) |
| Alcohol use disorder | 0.87 (0.70 - 1.07) |
| Substance use disorder | 1.10 (0.88 - 1.35) |
| Fall injuries | 0.78 (0.71 - 0.86) |
| Hospitalizations in the year before move-in | |
| 0-2 | 1.00 (ref.) |
| 3-5 | 1.24 (1.10 - 1.38) |
| ≥ 6 | 1.67 (1.49 - 1.88) |

**Supplementary Fig. 1**

Boxplot of temporary stay lengths by municipality. Each box represents the interquartile range (IQR), with the bottom and top edges indicating the first and third quartiles, respectively, and the midline indicating the median. The whiskers extend to the 10th and 90th percentiles


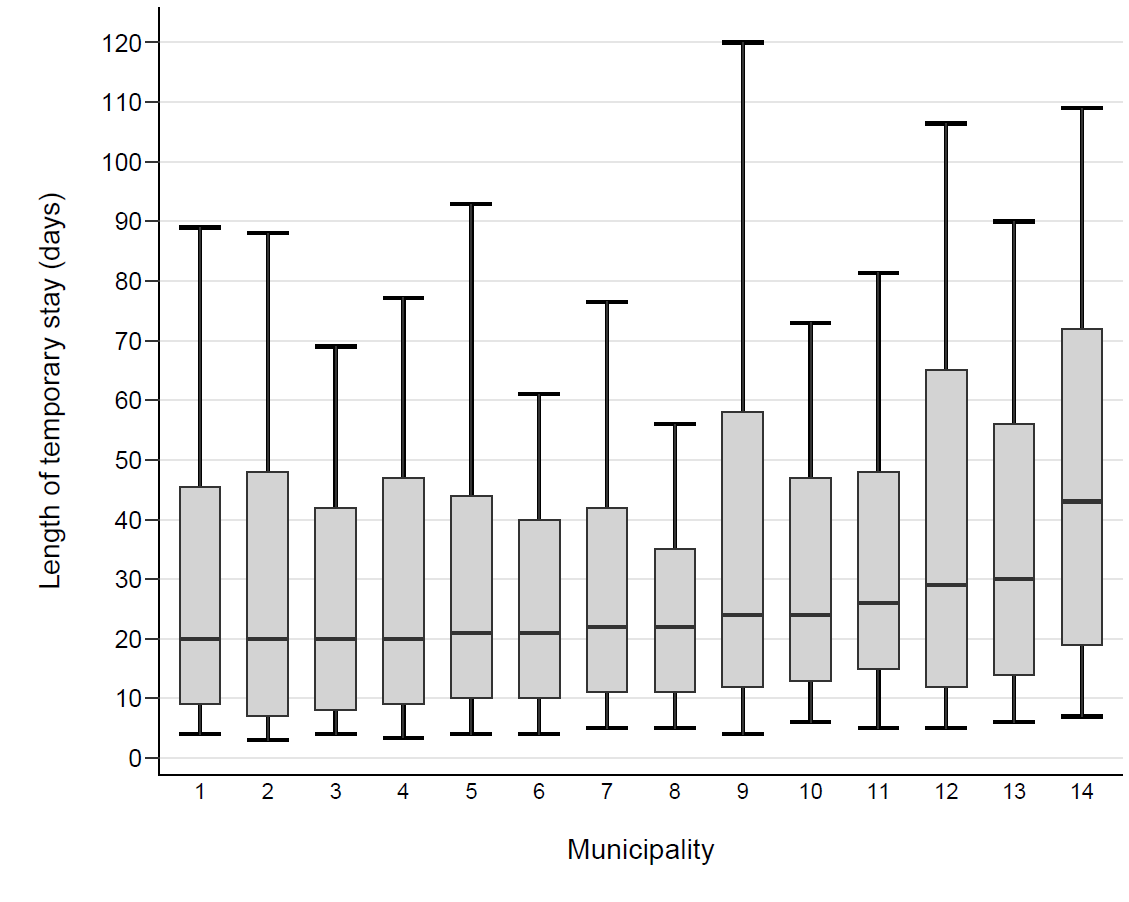


**Supplementary Fig. 2**

Distribution of hospital admissions by hour for patients admitted directly from temporary stay facilities


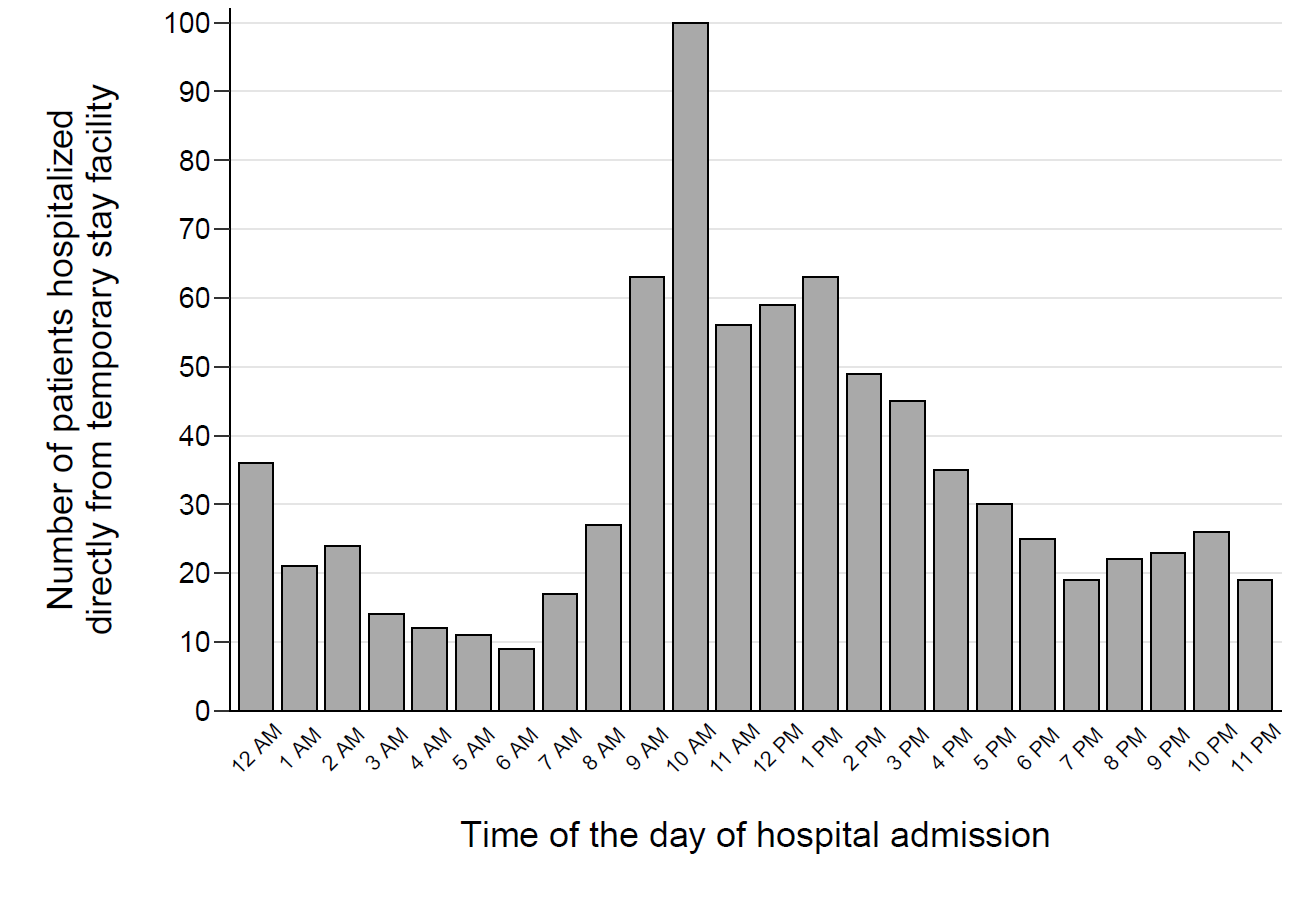


**Supplementary Fig. 3**

Distribution of hospital admissions by day of the week for patients admitted directly from temporary stay facilities


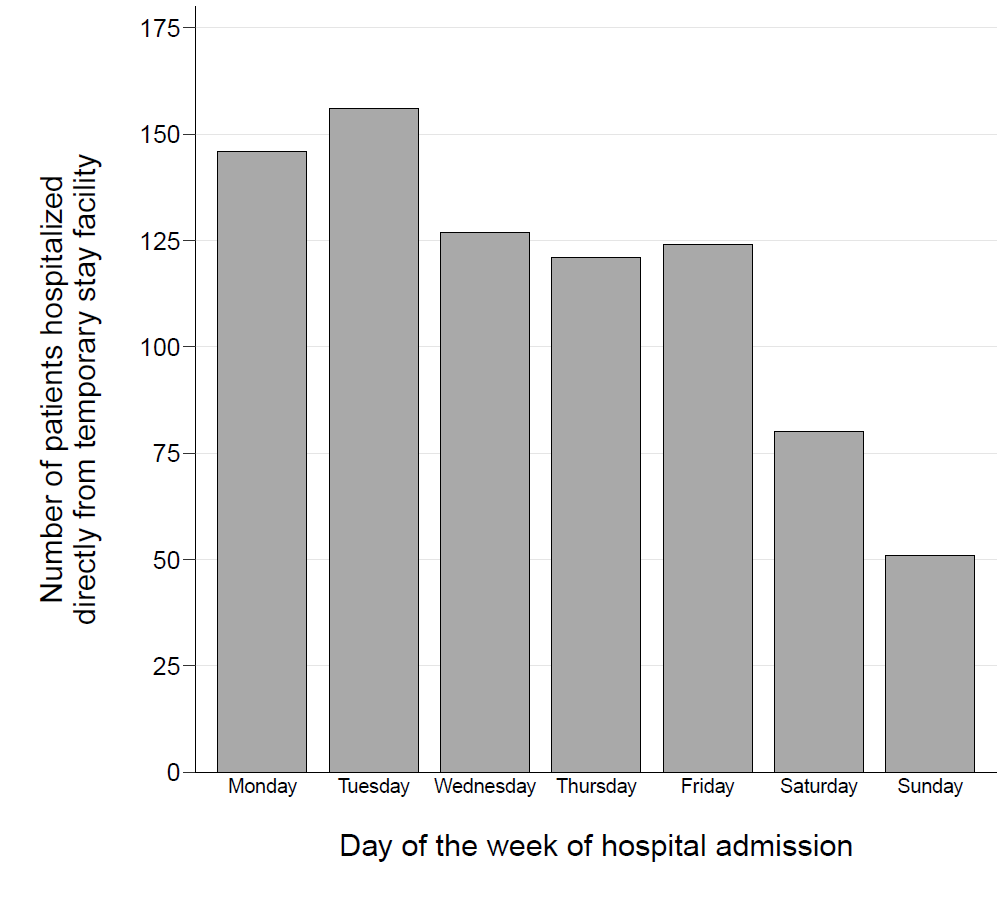


**Appendix A – Definition of comorbidities**

**A-table 1.** ICD-10 and ATC codes used to define comorbidities in Table 1

| **Comorbidity** | **ICD-10 code** | **ATC code** |
| --- | --- | --- |
| Cancer | C D45 D46 D471 D473 D474 D475 |  |
| COPD | J40 J41 J42 J43 J44 | R03 |
| Dementia | F00 F01 F02 F03 F1073 F1173 F1273 F1373 F1473 F1573 F1673 F1873 F1973 | N06D |
| Parkinson disease | F023 G20 G21 G22 | N04BA |
| Myocardial infarction | I20 I21 I22 I23 I24 I25 | N02BA C01DA B01AC |
| Heart failure | I099A I110 I130 I132 I50 | C03C |
| Atrial fibrillation | I48 |  |
| Stroke | I60 I61 I62 I63 I64 I69 |  |
| Diabetes mellitus | E10 E11 E13 E14 | A10 |
| Alcohol use disorder | F101 | N07BB |
| Substance use disorder | F11 F12 F13 F14 F15 F16 F17 F18 F19 | N07BC |
| Fall injuries | S00 S01 S02 S06 S10 S12 S20 S21 S22 S30 S31 S32 S40 S41 S42 S50 S51 S52 S70 S71 S72 S80 S81 S82 S430 S431 S432 S530 S531 |  |

*ICD-10 = 10^th^ revision of the International Classification of Disease; ATC = Anatomical Therapeutic Chemical; COPD = Chronic Obstructive Pulmonary Disease*
